# Transformation of a nonfunctional paraganglioma with I-123 MIBG scintigraphy correlation: A case report: Notice of Retraction

**DOI:** 10.1097/MD.0000000000005742

**Published:** 2016-12-16

The authors of the paper “Transformation of a nonfunctional paraganglioma with I-123 MIBG scintigraphy correlation: A case report”,^[1]^ which appeared in Volume 95, Issue 2 of *Medicine*, have asked that their case report be retracted. The patient that is the subject of the report was unavailable for consent and has since asked that it be removed. All images have been removed from the report.
